# The *Salmonella enterica* Plasmidome as a Reservoir of Antibiotic Resistance

**DOI:** 10.3390/microorganisms8071016

**Published:** 2020-07-08

**Authors:** Jean-Guillaume Emond-Rheault, Jérémie Hamel, Julie Jeukens, Luca Freschi, Irena Kukavica-Ibrulj, Brian Boyle, Sandeep Tamber, Danielle Malo, Eelco Franz, Elton Burnett, France Daigle, Gitanjali Arya, Kenneth Sanderson, Martin Wiedmann, Robin M. Slawson, Joel T. Weadge, Roger Stephan, Sadjia Bekal, Samantha Gruenheid, Lawrence D. Goodridge, Roger C. Levesque

**Affiliations:** 1 Institut de Biologie Intégrative et des Systèmes (IBIS), Université Laval, Québec, QC G1V 0A6, Canada; jean-guillaume.emond-rheault.1@ulaval.ca (J.-G.E.-R.); jeremie.hamel.1@ulaval.ca (J.H.); julie.jeukens.1@ulaval.ca (J.J.); l.freschi@gmail.com (L.F.); kukavica@fmed.ulaval.ca (I.K.-I.); brian.boyle@ibis.ulaval.ca (B.B.); 2 Health Canada, Bureau of Microbial Hazards, Ottawa, ON K1A 0K9, Canada; sandeep.tamber@canada.ca; 3 McGill Research Center on Complex Traits, McGill University, Montréal, QC H3G 0B1, Canada; danielle.malo@mcgill.ca; 4 Departments of Medicine and Human Genetics, McGill University, Montréal, QC H3G 0B1, Canada; 5 Centre for Infectious Disease Control (CIb), National Institute for Public Health and the Environment (RIVM), 3720 BA Bilthoven, The Netherlands; eelco.franz@rivm.nl; 6 Institute of Parasitology, McGill University, Montréal, QC H9X 3L9, Canada; elton.burnett@mail.mcgill.ca; 7 Département de Microbiologie, Infectiologie et Immunologie, Université de Montréal, Montréal, QC H3T 1J4, Canada; france.daigle@umontreal.ca; 8 Swine and poultry Infectious Diseases Research Center (CRIPA-FRQNT), Montréal, QC H3C 3J7, Canada; 9 National Microbiology Laboratory, Public Health Agency of Canada, Guelph, ON N1G 3W4, Canada; gitanjali.arya@phac-aspc.gc.ca; 10 Department of Biological Sciences, University of Calgary, Calgary, AB T2N 1N4, Canada; kesander@ucalgary.ca; 11 Department of Food Science, Cornell University, Ithaca, NY 14853-7201, USA; mw16@cornell.edu; 12 Department of Biology, Wilfrid Laurier University, Waterloo, ON N2L 3C5, Canada; rslawson@wlu.ca (R.M.S.); jweadge@wlu.ca (J.T.W.); 13 Institute for Food Safety and hygiene, University of Zürich, CH-8057 Zürich, Switzerland; stephanr@fsafety.uzh.ch; 14 Laboratoire de santé publique du Québec, Sainte-Anne-de-Bellevue, QC H9X 3R5, Canada; sadjia.bekal@inspq.qc.ca; 15 Department of Microbiology & Immunology, McGill University, Montréal, QC H3G 0B1, Canada; samantha.gruenheid@mcgill.ca; 16 Food Science Department, University of Guelph, Guelph, ON N1G 2W1, Canada

**Keywords:** plasmid, *Salmonella enterica*, antimicrobial resistance, long-read sequencing, hybrid assembly

## Abstract

The emergence of multidrug-resistant bacterial strains worldwide has become a serious problem for public health over recent decades. The increase in antimicrobial resistance has been expanding via plasmids as mobile genetic elements encoding antimicrobial resistance (AMR) genes that are transferred vertically and horizontally. This study focuses on *Salmonella enterica*, one of the leading foodborne pathogens in industrialized countries. S. enterica is known to carry several plasmids involved not only in virulence but also in AMR. In the current paper, we present an integrated strategy to detect plasmid scaffolds in whole genome sequencing (WGS) assemblies. We developed a two-step procedure to predict plasmids based on i) the presence of essential elements for plasmid replication and mobility, as well as ii) sequence similarity to a reference plasmid. Next, to confirm the accuracy of the prediction in 1750 *S. enterica* short-read sequencing data, we combined Oxford Nanopore MinION long-read sequencing with Illumina MiSeq short-read sequencing in hybrid assemblies for 84 isolates to evaluate the proportion of plasmid that has been detected. At least one scaffold with an origin of replication (ORI) was predicted in 61.3% of the *Salmonella* isolates tested. The results indicated that IncFII and IncI1 ORIs were distributed in many *S. enterica* serotypes and were the most prevalent AMR genes carrier, whereas IncHI2A/IncHI2 and IncA/C2 were more serotype restricted but bore several AMR genes. Comparison between hybrid and short-read assemblies revealed that 81.1% of plasmids were found in the short-read sequencing using our pipeline. Through this process, we established that plasmids are prevalent in *S. enterica* and we also substantially expand the AMR genes in the resistome of this species.

## 1. Introduction

Non-typhoidal *Salmonella enterica* is responsible for 88,000 cases of gastroenteritis in Canada each year. The symptoms of gastroenteritis can be mild to severe depending on the health conditions of individuals. Generally, the patient may recover without antibiotic treatment. However, antibiotic intervention may be necessary for children, the elderly, and immunosuppressed patients.

The *Salmonella* genus belongs to the *Enterobacteriaceae* family and includes two species, *bongori* and *enterica*. According to the Kauffman–White scheme, more than 2500 serotypes have been characterized [1]. As reported by the US Centers for Disease Control and Prevention (CDC), although all *S. enterica* serotypes can cause disease in humans, less than 100 serotypes account for much of the infections.

In 2014, a global report by the World Health Organization (WHO) on the surveillance of antimicrobial resistance (AMR) revealed that increasing resistance across many different infections has become a serious concern for public health worldwide [2]. AMR can be acquired by either spontaneous mutations or by horizontal gene transfer (HGT), in which plasmids are known to play a key role [3]. Plasmids are mobile genetic elements (MGE) encoding for their self-replication and transfer. The genes responsible for plasmid maintenance and transmission form a “backbone” that is a core set of genes encoding for essential plasmid functions [4]. Plasmids also provide non-essential cellular functions, such as virulence factors, AMRs, metabolic pathways, and unknown functions that are defined by genes encoding hypothetical and unknown proteins, which all confer competitive advantages to the bacterial host in specific situations. Once an AMR gene becomes stable on a plasmid through environmental pressures, it can quickly spread across species and ecosystems that can lead to its transfer from the surrounding environment to human pathogens [5,6].

Plasmids are amenable to detailed analysis using data from whole genome sequencing (WGS), using complementary software including mlplasmid, PlasmidFinder, cBar, plasmidSPAdes, Recycler, and PLACNET [7,8,9,10,11,12]. A recent comparison between five bioinformatics software showed that plasmidSPAdes was capable of fully or partially predicting 84% of plasmids used as references [12]. However, as plasmidSPAdes separates plasmids from chromosomes based on read coverage, plasmid contigs with a similar coverage to the chromosomal contigs are often mislabelled [8].

Plasmids are widespread in *S. enterica,* where they are known to carry nonessential genes involved in AMR and virulence [13,14]. Given that the spreading of AMR genes through microorganisms is a major issue for public health worldwide, the prediction of plasmid-carrying AMR genes will give insight into their dissemination across bacterial strains. In the current study, we expanded our knowledge of AMR genes carried by the plasmidome of 1750 *S. enterica* genomes. These genomes sequenced by Illumina MiSeq as part of a *Salmonella* Syst-OMICS project were analyzed by Plasmid-Gather, a pipeline designed to predict plasmid scaffolds based on the presence of essential genes for plasmid replication, mobility, and sequence similarity to a reference plasmid.

## 2. Materials and Methods

### 2.1. Bacterial Isolates and Growth Conditions

The *Salmonella enterica* isolates used in this study are described in Supplementary Table S1. The isolates were grown for 16–18 h at 37 °C on brain heart infusion agar (BHIA; Difco). The isolates were then transferred into Luria–Bertani (LB) broth with either 15% (*v/v*) glycerol or 8% (*v/v*) DMSO and stored at -80 °C until needed.

### 2.2. DNA Preparation and Sequencing

Genomic DNA was extracted from 1 mL of 4 mL LB broth cultures incubated for 16–18 h at 37 °C in agitation at 200 rpm, using the E–Z 96 Tissue DNA Kit (Omega Bio-tek, Norcross, GA, USA). Approximately 500 ng of genomic DNA was mechanically fragmented for 40 s with a Covaris M220 (Covaris, Woburn, MA, USA) using the default settings. Libraries were synthesized using the NEBNext Ultra II DNA library prep kit for Illumina (New England Biolabs, Ipswich, MA, USA) according to the manufacturer’s instructions and sequenced to obtain 30Xs coverage in an Illumina MiSeq 300 bp paired-end run at the Plateforme d’Analyses Génomiques of the Institut de Biologie Intégrative et des Systèmes (Université Laval, Québec, QC, Canada).

### 2.3. Library Preparation and Oxford Nanopore MinION Sequencing

Genomic DNA was extracted from 16-18 h LB broth cultures at 37 °C using the DNeasy Blood and Tissue Kit (QIAGEN, Toronto, ON, Canada). The manufacturer’s protocol has been adapted to maximize the read length (performed without any rapid pipetting, vortexing, and only homogenization by inversion). We used 1.2 µg of a genomic DNA for library preparation with the SQK-LSK109 Kit and followed the manufacturer’s recommendations. Native barcoding expansion PCR-free EXP-NBD104 (1–12) and EXP-NBD114 (13–24) kits were used according to the manufacturers’ protocol. Twenty-four libraries were pooled and sequenced with a R9.4.1 flow cell (FLO-MIN106D) on a MinION device. On average, one sequencing run gave between 20 to 28 Gb of raw data per run.

### 2.4. Bio-informatic Analysis and Databases

The origins of replication (ORIs) database (PlasmidFinder-DB) was downloaded from the PlasmidFinder web server [7] (accessed 23/10/2018 10:10). A homemade database (MOBs-DB) of proteins involved in plasmids conjugation, mobilization, or transfer was constructed with annotated proteins encoded by plasmids described in the UniProtKB database [15] (accessed 29/08/2017 20:48). The resulting plasmid mobility proteins were clustered by CD-HIT [16] using 60% and 85% amino acids identity as a cut-off and minimal alignment coverage for the longer and shorter sequences, respectively. By this process, the mobility proteins database contained 361 reference proteins. A plasmids database (Plasmids-DB), containing 13,924 plasmids on 23 October 2018, was downloaded from the National Center for Biotechnology Information (NCBI) (ftp://ftp.ncbi.nlm.nih.gov/genomes/refseq/plasmids/, accessed 23/10/2018 10:15). To ensure that the sequences in the Plasmids-DB were of plasmid origin and not mislabeled chromosomal DNA, we examined all the plasmids with more than 550 kb of DNA. We identified 2 sequences belonging to *Salmonella* plasmids with large sizes (NZ_CP022019.1 = 4,627 kb and NZ_LN868944.1 = 728 kb). NZ_CP022019.1 is most likely a complete chromosome mislabeled as a plasmid because it has the same size as a typical *Salmonella* chromosome. A search of the NCBI non-redundant nucleotide collection database (nr/nt) using the basic local alignement search tool (BLAST) revealed that NZ_LN868944.1 aligned completely with the *Salmonella* chromosomes; these 2 sequences were removed from the Plasmids-DB.

### 2.5. Plasmid-Gather Pipeline

**Figure 1:**
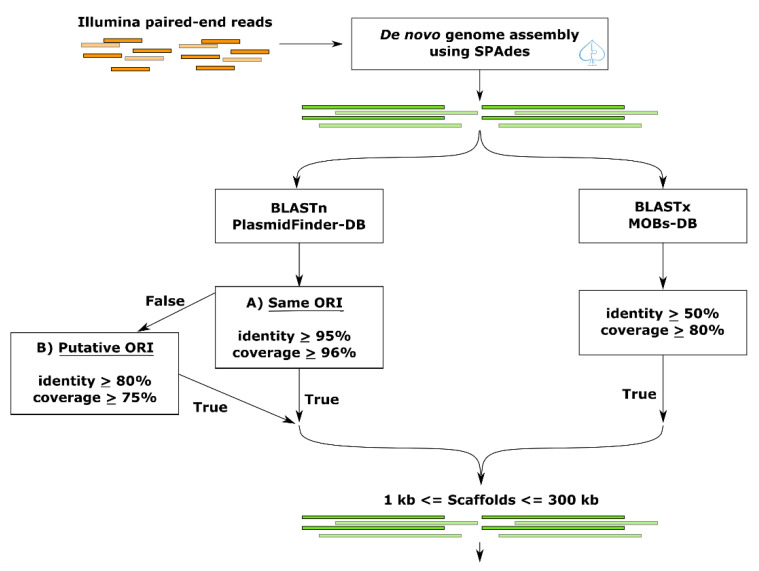
Plasmid-Gather workflow. First, MiSeq Illumina paired-end reads were trimmed using Trimmomatic (version 0.36) [17] and assembled by SPAdes (version 3.10.1) [18]. BLAST analysis (version 2.6.0) [19] against the origin of replications (ORIs) (PlasmidFinder-DB) and mobility proteins databases (MOBs-DB) were performed to predict which scaffolds carried one of these elements. The significant matches against the PlasmidFinder-DB were separated into two groups depending on the threshold: A) the highly similar ORIs with the database or B) the ORIs related to ORIs in the PlasmidFinder-DB. By the latter threshold, we wanted potentially to expand the discovery of plasmid scaffolds. Only scaffolds encoding an ORI and/or a plasmid mobility protein between 1 and 300 kb in size were kept for future analysis.

With the aim of detecting plasmids that can carry AMR or virulence genes, we developed an approach that combines 2 types of databases, thereby enhancing the discovery rate of contigs as plasmid fragments. The Plasmid-Gather pipeline is summarized in Figure 1. Briefly, reads were trimmed using Trimmomatic (v. 0.36) [17] and assembled by SPAdes (v. 3.10.1) [18]. We chose SPAdes as an assembler instead of plasmidSPAdes (an algorithm based on Bruijn graph to assemble plasmids), because the latter assembles only reads with coverage different from the chromosome, and therefore low copy plasmids with similar coverage to the chromosome are missed. SPAdes was used to obtain the whole genome assembly, and the plasmids were recovered based on two databases. BLAST (v. 2.6.0) [19] was performed on the resulting scaffolds against the PlasmidFinder-DB (see the above section on databases) to predict the ORI. Since it was expected that some plasmids assembled in several scaffolds were caused by repeated elements [20,21], we added a BLAST (v. 2.6.0) [19] search against a MOBs-DB (see the above section on databases) instead of using exclusively the PlasmidFinder-DB to maximize the discovery of contigs as plasmid fragments. To remove the possible chromosome contigs from the resulting scaffolds predicted by the pipeline, scaffolds with more than 300 kb were not taken in account. Another threshold to exclude sequences less than or equal to 1 kb was also included. To investigate whether the scaffolds corresponded to a known plasmid, the resulting scaffolds were aligned using BLAST (v. 2.6.0) [19] against the NCBI Plasmid database (≥ 95% sequence identity). The percentage of GC content was calculated using the infoseq application from the EMBOSS package (v. 6.5.7) [22].

### 2.6. Antibiotic Resistance Gene Analysis

AMR genes were predicted using Resistance Gene Identifier (RGI) (v. 4.2.2) based on the BLAST search against the Comprehensive Antibiotic Resistance Database (CARD) [23]. The presence of AMR genes was determined based on the curated e-value cut-offs.

### 2.7. SISTR

The *Salmonella* serotypes were predicted from the genome assemblies using the *Salmonella In Silico* Typing Resource (SISTR) (v. 1.0.2) [24].

### 2.8. Plasmid Fragments Recovery (Post-Recovery)

To increase the discovery rate in the Illumina MiSeq assembly of contigs as DNA plasmid fragments, we added an additional step in the Plasmid-Gather pipeline illustrated in Figure 1 to recover plasmid fragments without ORI or mobility proteins. For each scaffold with an ORI identified by Plasmid-Gather, we identified the plasmid with the highest bit score using BLAST against the NCBI Plasmid database (≥ 95% sequence identity, ≥ 15% query coverage per subject, and ≤ 550 kb of subject length). This plasmid, which varied with the plasmid scaffold containing an ORI(s) (query sequence), was used as a reference plasmid to align the Illumina MiSeq assembly. To be considered as a plasmid fragment, the scaffolds must share a ≥ 95% sequence identity and a ≥ 25% query coverage per subject with the reference.

## 3. Results

### 3.1. Construction of a Bioinformatics Pipeline for Plasmid Identification

In the framework of the Syst-OMICs genome project, 1750 *S. enterica* isolates (Supplementary Table S1) representing 153 serotypes analyzed by WGS (Supplementary Table S2) and available in the SalFoS database (https://salfos.ibis.ulaval.ca/) [25], were analyzed using the Plasmid-Gather pipeline depicted in Figure 1. Plasmid-Gather was developed to identify plasmid-related scaffolds in WGS data for *S. enterica* isolates by combining a systematic integrated strategy.

Plasmid-Gather identified 2211 scaffolds matching our criteria (i.e., scaffolds between 1 and 300 kb in size encoding either a plasmid mobility protein and/or an ORI) (Supplementary Table S3). There were three scaffolds > 300 kb (750, 515, and 421 kb) that were predicted to be plasmids based upon protein homology with sequences contained in the MOBs-DB (none encoded an ORI). A BLAST of these scaffolds against the NCBI Nucleotide collection (nr/nt) database revealed that they aligned with *Salmonella* chromosomes, but at 94%, 84%, and 94% of query cover, respectively. The genomic regions encoding the mobility proteins predicted by Plasmid-Gather were missed in these three alignments. A BLAST against the NCBI Nucleotide collection (nr/nt) database of these genomic regions where mobility proteins were identified revealed they corresponded to partial plasmid sequences. By evaluating this information, these three scaffolds could presumably be chromosomal DNA contigs carrying an integrated plasmid or an integrated conjugative element (ICE).

Scaffolds identified by Plasmid-Gather were labelled as “plasmid scaffolds”. Of these 2211 plasmid scaffolds, our pipeline predicted at least one ORI for 1910 of them. The GC-content, ORI type(s), and presence of the mobility protein are described in Supplementary Table S3. To provide an overview of the plasmid scaffolds that have already been characterized, the percentage coverage of the best alignments using BLAST (identity ≥ 95%) against the NCBI Plasmid database are presented in Supplementary Table S3.

We predicted from 1 to 10 plasmid scaffolds in 1097 *S. enterica* isolates. Of these 1097 isolates, one or several ORIs have been predicted in 1073 isolates. Some plasmids belonging to the *Enterobacteriaceae* were characterized and confirmed to contain more than one ORI [26]. We assume that possessing multiple ORIs would presumably allow plasmids the ability to replicate when transferred into another species or serotype and broaden the range of hosts. Overall, we predicted 2350 ORIs, of which 851 were termed as putative ORIs (Supplementary Table S4). Although the ORI termed as “putative ORI” contains the name of its closest BLAST match (e.g., putative-*IncFII*), putative ORIs were considered as a different type of ORI. Further analysis will be needed to consider a putative ORI as part of the same incompatibility group (Inc) to its closest BLAST match. A collection of 12 plasmid scaffolds carried three ORIs (10 IncFIB/IncFII/IncX1, 1 IncFIB/IncFII/IncI1, and 1 IncFIA/IncHI1A/IncHI1B), 416 had two ORIs (251 IncFIB/IncFII and 16 other combinations), and 1471 had one ORI. The three most frequent ORIs identified in *Salmonella* isolates, excluding the 851 putative ORIs, were the transferable IncFII (n_isolate_ = 533), IncFIB (n_isolate_ = 371), and IncI (n_isolate_ = 142) (Table 1). All three Inc group plasmids have been shown to carry virulence-associated and AMR genes within *Enterobacteriaceae* [14,27,28,29,30]. The majority of the IncFII and IncFIB ORIs were identified from *S.* Enteritidis and *S.* Typhimurium (Table 1) (at 33% and 28% for IncFII and at 47% and 39% for IncFIB, respectively). Moreover, IncFII was co-carried with IncFIB in 97% of the isolates (359/371). Even putative ORIs were excluded in Table 1; the three most widespread ORIs between *Salmonella* serotypes were IncFII (n_serotype_ = 50), IncI1 (n_serotype_ = 43), and ColpVC (n_serotype_ = 37). In contrast, the two ORIs, IncX1 (n_serotype_ = 10) and IncA/C2 (n_serotype_ = 12), were limited to few serotypes (Table 1).

### 3.2. Antibiotic Resistance Genes of the S. enterica Plasmidome

**Figure 2:**
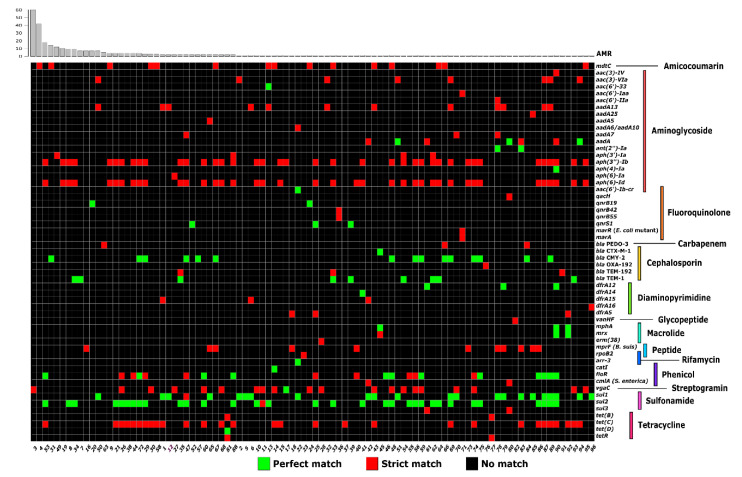
Antimicrobial resistance (AMR) genes of the *S. enterica* plasmidome predicted using the Resistance Gene Identifier (RGI) (v. 4.2.2), based on the Comprehensive Antibiotic Resistance Database (CARD) [23]. The bar plot above shows the frequency of unique resistomes. Numbers below the heatmap indicate the antimicrobial resistance profile (AMRp) (Supplementary Table S5). The AMRps were assigned to the resistome of each isolate plasmidome. The antibiotic family or function are shown on the right.

To evaluate the diversity of plasmid-encoded AMR genes that can potentially complicate disease treatments and potentially be transferred to other bacteria, we next predicted AMR genes carried by the *S. enterica* plasmidome using the Resistance Gene Identifier (RGI v. 4.2.2) [23]. The RGI predicted 863 AMR genes across 375 plasmid scaffolds in 327 *S. enterica* genomes (Supplementary Table S5). Plasmid scaffolds encoded 55 different AMR genes, and 96 unique resistomes encoded by plasmid scaffolds were found across the 327 genomes (Figure 2).

The plasmid scaffolds with the highest number of AMR genes were a single without ORI (absent) (n_AMR_ = 9), three containing IncA/C2 (n_AMR_ = 8), and one with IncL/M (n_AMR_ = 7). We found that the three most frequent ORIs carrying the AMR gene(s) were IncI1 (n_ORI_ = 98), followed by IncFII (n_ORI_ = 50) and IncX1 (n_ORI_ = 25). IncI1 has previously been the most common ORI type identified in multi-drugs resistance isolates [31]. However, in our studies plasmid scaffolds without an ORI are the second most common AMR gene carriers (86 scaffolds into 76 isolates). These are likely DNA fragments of larger plasmids of which the ORI assembled on another scaffold. Plasmids can be assembled in several scaffolds because of repeated elements. Further analysis using PCR or DNA long-read sequencing will be required to order scaffolds for assembly.

AMR genes were also identified for the 1750 *S. enterica* genomes in which plasmid scaffolds were removed and both resistomes were compared (Supplementary Table S6). The prevalence of AMR genes available from the CARD website (v. 4.2.2) among *Salmonella* genomes from the NCBI Chromosome and NCBI Plasmid databases were included in Supplementary Table S6 for comparative purposes. An analysis of 1750 *Salmonella* chromosomes showed an average of 39 AMR genes by genome (from 30 to 56 AMR genes/ genome), which represented 207 different AMR genes. Thirty-four AMR genes were predicted in nearly all chromosomes (from 95 to 100% of chromosomes) (26 out of these 34 resistance mechanisms belong to an antibiotic efflux pump complex) (Supplementary Table S6). These genes may likely correspond to the core resistome of *S. enterica*. Moreover, the prevalence of 25 of these AMR genes in the NCBI Plasmid and Chromosome databases found in the CARD database were consistent with our prediction (Supplementary Table S6). Overall, 17 AMR genes that were infrequently predicted in chromosomes are normally limited to the NCBI Plasmid database according to CARD (0% in NCBI Chromosome database) (Supplementary Table S6), which suggests that some plasmid scaffolds may remain unidentified among chromosome scaffolds.

### 3.3. Increasing Recovery of Plasmid Scaffolds Using a Reference

**Figure 3:**
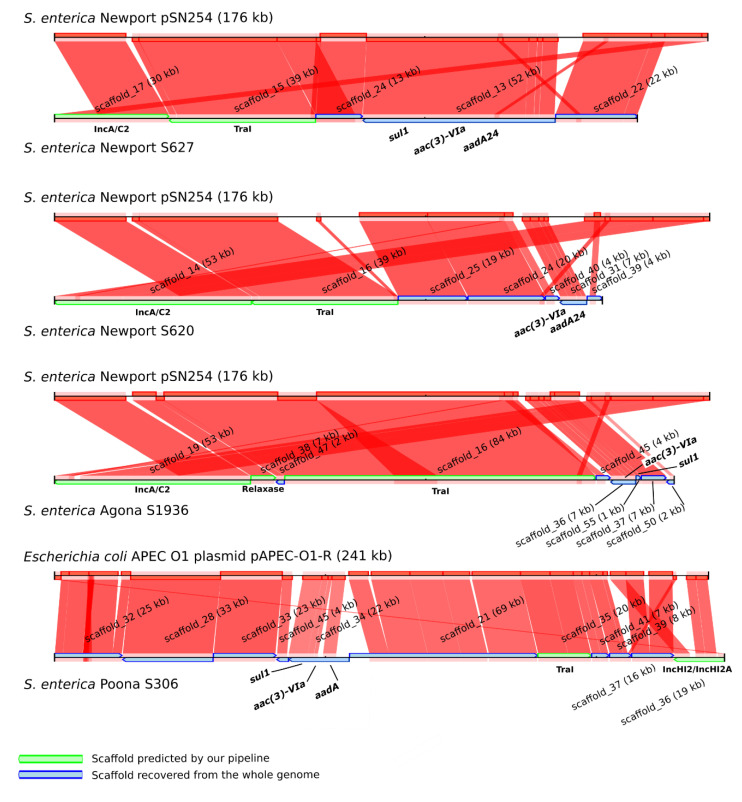
Recovery of plasmid fragments in whole genome sequencing (WGS) data based on comparisons with reference plasmids. The reference plasmids above each alignment were selected based on the best match against the National Center for Biotechnology Information (NCBI) Plasmid database with the scaffold encoding *aac(3)-VIa* gene usually found in plasmid sequences. The WGS data were mapped using CONTIGuator v. 2.7 against the reference plasmids. Several scaffolds without ORI or mobility protein have been identified as plasmid scaffolds in the four *Salmonella* genomes using a reference plasmid.

To investigate whether plasmid scaffolds remained within the *S. enterica* chromosome scaffolds, the four chromosomal scaffolds carrying *aac(3)-VIa*, a resistance gene limited to the NCBI Plasmid database, were aligned against the NCBI Plasmid database, and their best match was used as reference to map the Illumina MiSeq assemblies (Figure 3). 

In three of the four cases, the best match from the NCBI Plasmid database was the multidrug-resistant plasmid IncA/C pSN254, while the fourth was identified as IncHI2 pAPEC-01-R [32,33]. To confirm the ORI type of the plasmids from Plasmids-DB, we aligned all the plasmids of the NCBI Plasmid database using BLAST against the PlasmidFinder-DB [7]. The plasmid pSN254, as well as many *Salmonella* plasmids first published as IncA/C (pAM04528, peH4H, pAR060302, p1643_10, p33676, pCVM2245, pCVM22462, pCVM22513, pCVM21538, pCVMN1543, and pCVM21550), perfectly matched with IncA/C2; meanwhile, pRA1 and pRAx matched with IncA/C [34,35,36,37,38]. As mentioned by Carattoli et al. (2006), IncA/C and IncA/C2 exhibit 26 nucleotide substitutions [39]. As demonstrated in Figure 3, at least two scaffolds that aligned with the reference plasmid had already been detected in the WGS using our pipeline; one encoded the TraI mobility protein, whereas the second carried the ORI(s) (IncA/C2 or IncHI2/IncHI2A). Furthermore, as depicted in Figure 3, several new plasmid scaffolds were recovered using the closest homologous plasmid from NCBI as the reference plasmid. Figure 3 also shows the complexity of plasmid reconstruction, which is probably due to the high plasticity of some plasmids, as observed for IncHI2 and IncA/C [31,40,41].

To improve the detection of plasmid fragments, we added a last step that used a reference plasmid to the Plasmid-Gather pipeline, which is illustrated in Figure 1. However, instead of using the scaffold encoding the AMR gene to select the closest reference plasmid, we picked the reference based on the best match with the scaffold bearing the ORI. We recovered 1172 scaffolds for the *S. enterica* plasmidome, giving a total of 3383 scaffolds. By taking this data into account, the scaffolds were re-assorted into two groups as plasmid and chromosome scaffolds.

### 3.4. The Resistome of S. enterica Plasmids and Chromosomes

We predicted AMR genes using RGI v. 4.2.2 [23], but in the *S. enterica* plasmid and chromosome scaffolds that were separated using the strategy that we called post-recovery. The AMR genes predicted in either the post-recovery plasmid or chromosome scaffolds of *S. enterica* showed a better AMR gene specificity carried by each. RGI predicts in plasmids a total of 1174 AMR genes (311 new AMR comparing with the previous prediction). Several predicted AMR genes, which are frequently found in the NCBI Plasmid database when compared to the NCBI Chromosome database, showed similar distributions with CARD (Supplementary Table S7). For instance, before the recovery of plasmid scaffolds, 36 and 37 *bla*_CMY-2_ genes were predicted in plasmids and chromosomes, respectively (Supplementary Table S6). After the post-recovery strategy based on a reference plasmid, 69 (6.26%) and 4 (0.23%) *bla*_CMY-2_ were identified within plasmids and chromosomes (Supplementary Table S7). The prevalence of the *bla*_CMY-2_ gene in the *S. enterica* genomes from the NCBI Plasmid and NBCI Chromosome databases are 6.62% and 0.73%, respectively, which is consistent with what we obtained post-recovery (Supplementary Table S7). A similar trend was observed for the *sul1*, *bla*_TEM-1_, *tet(B), tet(C)*, *tet(D), tetR,* and *aph(3’)-Ia* AMR genes.

To determine whether there is an enrichment of certain AMR genes between the plasmids and chromosomes of *S. enterica*, we calculated *p* values using Fisher’s exact test (Supplementary Table S7). We noted that 52 and 13 AMR genes were significantly enriched (*p* < 0.001) in chromosomes and plasmids, respectively (Supplementary Table S8 and Table 2).

### 3.5. Analysis of Plasmid Content Using Long-Read DNA Sequencing 

**Figure 4:**
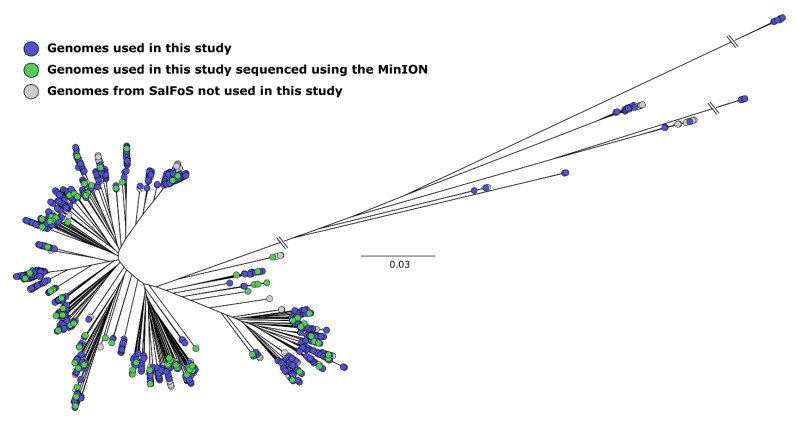
Unrooted maximum likelihood tree of 2544 *S. enterica* genomes based on 173,657 single nucleotide polymorphisms (SNPs). Genomes were assembled using SPAdes. Isolates used are labelled in green and blue. Green nodes were isolates sequenced using Oxford Nanopore.

As depicted in Supplementary Table S9, 84 *Salmonella* isolates were selected from distant branches of a phylogenetic tree representing 2544 *S. enterica* genomes to get the inter alia maximum genome and plasmid diversity contained in the *S. enterica* species (Figure 4). Additionally, expanding the WGS using the Oxford Nanopore giving DNA long-reads and combining this data with Illumina MiSeq short-reads in the hybrid genome assembly of the complete chromosome and plasmid contents allowed us to evaluate our prediction in the short-reads data using Plasmid-Gather.

The combined hybrid assemblies gave the complete bacterial chromosome in all the 84 isolates selected. Hybrid assemblies of the complete chromosomes also indicated 73 scaffolds, of which 64 were predicted as plasmids (87.7%) using the ORIs (PlasmidFinder-DB) and the mobility proteins (MOBs-DB) databases that predicted plasmids in the short-read assemblies (Supplementary Table S9). Mobility proteins or ORI sequences were not identified in the *Salmonella* chromosomes, demonstrating a high specificity of the two databases for extrachromosomal elements. The percentage of each plasmid assembled using hybrid assemblies covered by those predicted in the MiSeq data has been calculated, and the sum showed that 81.1% of plasmids were found in the short-read assemblies. Nine extrachromosomal elements, of which eight are small elements (less than 7 kb), could not be identified as plasmids in both assemblies, although the online BLAST searches on NCBI indicated that they matched with the plasmid sequences.

The AMR genes were predicted using RGI v. 4.2.2 in the 73 extrachromosomal elements only [23] (Supplementary Table S9). This analysis gave two major observations: 1) the most frequent ORI carrying AMR genes was IncI1 (4/5), and 2) the plasmid with the greatest number of AMR genes was an IncA/C2 (n_AMR_ = 12; *aac(3)-VIa, aph(3’’)-Ib, aph(6)-Id, bla*_CMY-35_*, bla*_CMY-44_*, bla*_CMY-80_*, bla*_CMY-90_*, aadA13, floR, sul1, sul2* and *tet(C))* (Supplementary Table S9). Both observations were consistent with our prediction in the MiSeq data described above.

## 4. Discussion

By combining a collection of public and SalFoS data, we identified a high proportion of plasmid contigs in Illumina MiSeq WGS assemblies using two databases containing essential conserved plasmid elements (PlasmidFinder-DB and MOBs-DB) combined with known reference plasmids. One of the added values will be to increase the plasmid sequences identified. The databases can be regularly updated to include new ORIs and mobility genes for future analyses.

By using Plasmid-Gather and the combined strategies described here, IncFII and IncFIB were the most frequent ORIs predicted in *S. enterica*; this was presumably caused by the over-representation of the *S.* Enteritidis and *S.* Typhimurium isolates in the dataset (Table 1). These two serotypes accounted for more than 23% of all the isolates and carried together 61% and 87%, respectively, of all th eIncFII and IncFIB (Table 1). The distribution of ORIs among the *S. enterica* serotypes showed that IncFII, IncI1, and ColpVC were found in a broad range of serotypes, whereas IncA/C2 and IncX1 are restricted to a dozen serotypes (Table 1). Interestingly, Lindsey et al. (2009) demonstrated by a cluster-based analysis using the pulsed field gel electrophoresis (PFGE) of 216 multidrug resistance *S. enterica* that IncI1 is not clonally distributed, whereas IncA/C is commonly observed in the same serotypes. Hence, IncI1 is presumably much more mobile than IncA/C [42]. IncI1 incompatibility was often associated with multi-drug resistance and with the widespread distribution of Beta-lactam resistance genes [28,29,41,43]. Likewise, we observed that plasmids with IncI1 are among the most important carriers of AMR genes (Supplementary Table S5 and Supplementary Table S9). Hence, one may assume that the mobility of IncI1 also leads to the spread of AMR genes in many *S. enterica* serotypes, whereas IncA/C2 seems more serotype restricted, but were associated with several AMR genes.

Large plasmids, representing different Inc ORIs, are known to integrate and carry transposons or integrons conferring AMR [3,14]. Several multi-resistance plasmids have been identified in *Salmonella*. Among them is the Inc group A/C (IncA/C and A/C2), consisting of 150 kb plasmids [31,33,38,42,44]. In our study, 49 of the 53 IncA/C2 plasmid scaffolds had less than 54 kb. This may be due to the limitations of plasmid assembly, as demonstrated in Figure 3. Three of the four IncA/C2 assembled with expected sizes encoding seven AMR genes each (*aph(3*’’)*-Ib*, *aph(6)-Ib*, *bla*_CMY-2_, *sul1*, *sul2*, *aad* or *aad7,* and *florR* or *aac(3)-IV*) (Supplementary Table S5). In addition, the IncA/C2 plasmid reconstructed by hybrid assembly from the S624 isolate possessed 12 AMR genes, a greater number than the other plasmids carrying AMR genes obtained using hybrid assemblies (Supplementary Table S9). Furthermore, in the Illumina MiSeq data we noted that nearly all the *S. enterica* isolates with IncA/C2 (52/53) had one or more AMR encoded by scaffolds in their plasmidome. Isolates with IncA/C2 plasmids carried on average five AMR genes (up to 11 for the S628 isolate); IncA/C2 is the only ORI predicted in 23/53 genomes. Multidrug resistance isolates have been linked previously to IncA/C [42].

Regarding the 52 AMR genes significantly enriched amongst *S. enterica* chromosomes, 34 were part of what we call "the core resistome"—i.e., AMR genes found in more than 95% of *S. enterica* genomes (described in Supplementary Table S8). The predominant AMR mechanism in the so-called core resistome is antibiotic efflux (26/34). Efflux transporters exist as either single- (e.g., Tet) or multi-component pumps (e.g., MdsABC complex) [45]. Multidrug efflux pumps are common resistance mechanisms among Gram-negative bacteria [45]. However, due to various efflux pumps that can compensate with wide substrate specificity, it remains a challenge to identify which drug efflux pump confers AMR. For other less frequent AMR genes found in *S. enterica*, *aac(6’)-Ia,a* and *aac(6’)-Iy* sharing a 99% amino acid identity were found in different serotypes (e.g., Typhimurium, Braenderup, and I 4 [5];12;i;- for *aac(6’)-Iaa*; Enteritidis, Newport, and Heidelberg for *aac(6’)-Iy*). Together, these 2 *N*-acetyltranferases (AAC) were encoded within 1738 chromosomes (99.3%), as shown in Supplementary Table S8. The gene *fosA7*, conferring resistance to fosfomycin, was predicted in 100% of the *S.* Heidelberg (51/51) isolates. In alignment with these results, fosfomycin resistance has previously been found in *S.* Heidelberg isolated from broiler chickens [46]. Similarly, *fosA7* was observed predominantly in almost all the isolates from the same serotype: in 96% of *S.* Agona (25/26), in 100% of the *S.* Telelkebir (8/8), in 67% of the *S.* Derby (8/12), and in 70% of the *S.* Alachua (7/10). The remaining *fosA7* genes (*n* = 23) were distributed among 14 under-represented serotypes.

Although considered as chromosome encoded, some efflux pumps have been identified on plasmids, such as the *tetA* gene encoding tetracycline resistance [47]. As shown in Table 2, 3 of the 13 AMR genes enriched in the *Salmonella* plasmidome encoded efflux pumps, 2 conferred resistance to tetracycline (*tet(C)* and *tet(D)*) and the last one was the resistance to chloramphenicol/florfenicol (*floR*). Tetracycline has been overused in human and veterinary medicines as growth promoters in animals [48,49]. The *tet(C)* and *tet(D)* AMR genes were often reported on MGEs, such as genomic islands (GEIs), as part of conjugative elements and in plasmids [50,51,52,53,54]. We observed a low abundance of *tet(C)* and *tet(D)* in isolates carrying plasmid scaffolds (7.9% and 2.2%, respectively) (Table 2). Previous studies have shown the rare occurrence of these AMR genes in *Salmonella enterica* strains [55,56]. The serotypes of *S. enterica* from SalFoS harboring *tet* genes were mostly *S.* Newport (29.9%, *n* = 26) and *S.* Typhimurium (23%, *n* = 20) for *tet(C)* and *S.* Kentucky (41.7%, *n* = 10) for *tet(D)*. The *floR* gene is the only significant plasmid gene conferring resistance to chloramphenicol (Table 2). We also noticed that 92.3% of the plasmidomes coding for *floR* also carried an IncA/C2, thereby leading to the conclusion that this ORI is likely to be strongly associated with its dissemination. The connection between *floR* and IncA/C2 can also be seen in hybrid assemblies, because *floR* was only predicted once in an IncA/C2 plasmid (Supplementary Table S9). The *floR* gene was already highlighted as the most common in *Salmonella* chloramphenicol-resistant strains [56,57]. 

In examining the AMR genes detected in plasmids (Table 2), the most common resistance encoded was resistance to streptomycin (*strA* and *strB* resistance genes). In addition to being used for human medicine, streptomycin is used as a feed supplements for pigs and as a pesticide for agriculture [48,58]. Likely because it is extensively used in agriculture, resistance to streptomycin was frequently found in environmental and pathogenic isolates [59,60]. Moreover, aminoglycoside antibiotic was the most prevalent drug class identified.

In this study, we were also interested in AMR genes that may complicate the treatment of salmonellosis and cause possible public health issues by the HGT of AMR genes. In 2014, of all antimicrobials prescribed in human medicine used for treating bacterial infections, the beta-lactam amoxicillin represented the largest proportion used (26%), followed by azithromycin (9%) and ciprofloxacin (8%) [61]. In the same year, 5% of the non-typhoidal *Salmonella* isolates were resistant to amoxicillin, while no resistance to azithromycin or ciprofloxacin was observed; these last two antimicrobials were largely prescribed to treat severe and invasive salmonellosis [61]. Three AMR genes identified in the *S. enterica* plasmidome confer resistance to either amoxicillin (*bla*_TEM-1_ (*n* = 47)), azithromycin (*mphA* (*n* = 4)) or ciprofloxacin (*aac(6’)-Ib-cr* (*n* = 1)) (Supplementary Table S7); the last two antibiotics are used to treat severe and invasive *Salmonella* infections [62,63,64]. Fortunately, these three AMR genes are infrequent in the *S. enterica* plasmidome, except for *bla*_TEM-1_, and are not co-carried by the same isolate. However, once an AMR gene is plasmid-stable, AMR resistance can quickly spread through bacterial communities, and so this is something that may need future monitoring. In contrast, there is no clear pattern among *S. enterica* isolates harboring *bla*_TEM-1_; these strains were isolated from 1981 to 2011 in five countries from eight species representing 15 *Salmonella* serotypes. Furthermore, nine different ORIs were found to be associated with scaffolds carrying *bla*_TEM-1_.

## 5. Conclusions

Dealing with the increasing multi-resistance of *S. enterica* isolates remains a major worldwide challenge. Over the last decade, mobile genetic elements including plasmids have contributed to the spread of AMR genes vertically and horizontally between serotypes. *S. enterica*, one of the leading foodborne pathogens in industrialized countries, is known to carry plasmids encoding AMR and virulence. We present an integrated strategy to identify plasmid scaffolds using WGS. We combined two databases containing essential elements for plasmid DNA replication (PlasmidFinder-DB) and for plasmid mobility (MOBs-DB). In the current study, we highlight the great diversity of plasmids present in *S. enterica* as reflected on the basis of ORIs diversity. Plasmids were identified in 1750 *S. enterica* genomes, representing 153 serotypes, and 61.3% of the genomes from 1073 of 1750 WGS data had at least one plasmid carrying an ORI, thereby confirming plasmid prevalence in *S. enterica*. Whereas the databases from NCBI, EMBL, and DDBJ are overflowing with WGS data, this is not significantly informative without metadata and the availability of isolates for future functional studies. The SalFoS *Salmonella* database was constructed for the public distribution of isolates for functional studies and serves as a convenient resource to accomplish the expansion of the metadata. 

## Figures and Tables

**Table 1:** Nine most frequent ORIs found across the most frequent *S. enterica* serotypes.

Serotype	Number of Isolates (Given by SISTR V. 1.0.2)	Nine Most Frequent ORIs (if n_replicon_ ≥ 20)								
		IncFII	IncFIB	IncI1	IncX1	ColpVC	IncA/C2	Col156	IncHI2A	IncHI2
Typhimurium	201	147	145	28	3	7	11	6	-	-
Enteritidis	200	179	178	9	24	3	-	1	-	-
Newport	80	14	-	2	3	-	25	-	1	1
Heidelberg	51	-	-	13	35	19	1	2	4	4
Oranienburg	49	30	-	-	-	1	-	-	-	-
Thompson	44	2	-	-	-	3	-	-	-	-
Muenchen	43	4	2	3	-	-	-	-	-	-
Infantis	38	-	-	5	-	-	1	-	-	-
Anatum	38	13	-	5	-	1	3	-	1	1
Senftenberg	36	-	1	-	-	-	-	-	-	-
Braenderup	34	3	3	-	-	1	-	-	-	-
Javiana	28	2	-	2	-	-	-	-	-	-
Saintpaul	26	4	1	3	-	2	-	1	2	2
Agona	26	1	-	7	-	1	1	-	1	1
I_4 [5]; 12:i:-	25	17	17	6	-	1	1	-	1	1
Montevideo	24	1	-	1	1	-	-	-	-	-
Paratyphi_B_var._Java_monophasic	23	16	-	1	-	1	-	-	-	-
Give	23	-	-	1	-	1	-	1	-	-
Gaminara	21	-	-	-	-	-	-	-	-	
Paratyphi_B_var._Java	20	1	-	3	-	1	-	-	-	-
Paratyphi_B	20	-	-	2	-	-	-	-	-	-
Kentucky	20	9	9	11	13	1	-	1	3	3
Typhi	17	-	1	-	-	-	-	-	-	-
Hartford	17	17	-	-	-	1	-	-	-	-
Tennessee	16	-	-	1	-	-	1	1	1	1
Mississippi	16	1	-	1	-	-	-	-	-	-
Mbandaka	16	-	1	-	-	1	-	-	1	1
Rubislaw	15	2	-	-	2	5	-	3	-	-
Manhattan	15	-	-	2	-	1	-	-	-	-
Dublin	15	15	-	-	15	-	5	-	-	-
Total of isolates	1197	478	358	106	96	51	49	16	15	15
Total of isolates in other serotypes	553	55	13	36	2	37	4	13	9	9
Total of isolates in all serotypes	1750	533	371	142	98	88	53	29	24	24
Count of serotypes	153	50	18	43	10	37	12	16	17	17

**Table 2:** Additional antimicrobial genes found in the *S. enterica* plasmidome.

AMR Genes with a *p*-Value ≤ 0.001	Prevalence (%)	Resistance Mechanism	Drug Class	Confers Resistance to
*aac(3)-Via*	1.4	antibiotic inactivation	aminoglycoside antibiotic	gentamicin B and C
*aph(3’’)-Ib (strA)*	10.5	antibiotic inactivation	aminoglycoside antibiotic	streptomycin
*aph(6)-Id (strB)*	10.4	antibiotic inactivation	aminoglycoside antibiotic	streptomycin
*aadA8*	0.8	antibiotic inactivation	aminoglycoside antibiotic	streptomycin and spectinomycin
*aadA13*	2.5	antibiotic inactivation	aminoglycoside antibiotic	streptomycin and spectinomycin
*bla*_CMY-2_	6.3	antibiotic inactivation	cephamycin and cephalosporin	cefoxitin, cephamycin and ceftazidime
*bla*_TEM-1_	4.3	antibiotic inactivation	penem, cephalosporin, monobactam, penam	amoxicillin, ampicillin and cefalotin
*floR*	4.7	antibiotic efflux	phenicol antibiotic	chloramphenicol and florfenicol
*sul2*	8.5	antibiotic target replacement	sulfonamide antibiotic, sulfone antibiotic	sulfadiazine, sulfadimidine, sulfadoxine, sulfamethoxazole, sulfisoxazole, sulfacetamide, mafenide, sulfasalazine and sulfamethizole
*tet(C)*	7.9	antibiotic efflux	tetracycline antibiotic	tetracycline
*tet(D)*	2.2	antibiotic efflux	tetracycline antibiotic	tetracycline
*mprF* (*Brucella suis*)	2.1	antibiotic target alteration	peptide antibiotic	defensin
*qnrB19*	0.7	antibiotic target protection	fluoroquinolone antibiotic	-

## Supplementary Materials

The following are available online at https://www.mdpi.com/2076-2607/8/7/1016/s1, **Table S1**: Isolates, **Table S2:** Serotypes, **Table S3:** Put. Plasmids, **Table S4:** Replicons Count, **Table S5:** AMR Profiles, **Table S6:** AMR Prelevance, **Table S7:** AMR Post-Recov, **Table S8:** AMR Chromosome, **Table S9:** Hybrid Assemblies.
